# Current status and prospect of PET-related imaging radiomics in lung cancer

**DOI:** 10.3389/fonc.2023.1297674

**Published:** 2023-12-18

**Authors:** Xin Tang, Fan Wu, Xiaofen Chen, Shengli Ye, Zhongxiang Ding

**Affiliations:** ^1^Department of Radiology, Hangzhou Wuyunshan Hospital (Hangzhou Health Promotion Research Institute), Hangzhou, China; ^2^Department of Nuclear Medicine and Radiology, Shulan Hangzhou Hospital affiliated to Shulan International Medical College of Zhejiang Shuren University, Hangzhou, China; ^3^Department of Radiology, Hangzhou First People’s Hospital, Hangzhou, China

**Keywords:** lung cancer, radiomics, positron emission tomography/magnetic resonance imaging, positron emission tomography/computed tomography, review

## Abstract

Lung cancer is highly aggressive, which has a high mortality rate. Major types encompass lung adenocarcinoma, lung squamous cell carcinoma, lung adenosquamous carcinoma, small cell carcinoma, and large cell carcinoma. Lung adenocarcinoma and lung squamous cell carcinoma together account for more than 80% of cases. Diverse subtypes demand distinct treatment approaches. The application of precision medicine necessitates prompt and accurate evaluation of treatment effectiveness, contributing to the improvement of treatment strategies and outcomes. Medical imaging is crucial in the diagnosis and management of lung cancer, with techniques such as fluoroscopy, computed radiography (CR), digital radiography (DR), computed tomography (CT), magnetic resonance imaging (MRI), positron emission tomography (PET)/CT, and PET/MRI being essential tools. The surge of radiomics in recent times offers fresh promise for cancer diagnosis and treatment. In particular, PET/CT and PET/MRI radiomics, extensively studied in lung cancer research, have made advancements in diagnosing the disease, evaluating metastasis, predicting molecular subtypes, and forecasting patient prognosis. While conventional imaging methods continue to play a primary role in diagnosis and assessment, PET/CT and PET/MRI radiomics simultaneously provide detailed morphological and functional information. This has significant clinical potential value, offering advantages for lung cancer diagnosis and treatment. Hence, this manuscript provides a review of the latest developments in PET-related radiomics for lung cancer.

## 1 Introduction

Lung cancer is a highly aggressive form of tumors. According to the Global Cancer Report 2020, there were 19.29 million new cancer cases worldwide, with lung cancer accounting for 2.2 million (11.4%), ranking it second only to breast cancer (11.7%). In 2020, cancer-related deaths fatalities 9.96 million, of which 1.8 million (18%) were attributed to lung cancer, making it a leading cause of cancer-related mortality. Particularly affecting men, lung cancer holds the top position in both incidence and mortality among male patients (1, 2). The 5-year survival rates after surgery vary significantly among lung cancer patients at different stages. Stage IIIA~IVA patients have survival rates as low as 10%~36%, whereas stage I patients may achieve survival rates ranging from 77% to 92% (3). Consequently, early detection emerges as the crucial factor in reducing patient mortality (1–4). Although there has been some progress in lung cancer survival rates in recent decades, the overall 5-year survival rate remains low, typically ranging between 10% and 20% (2). Personalized treatment represents the pivotal approach to enhancing survival rates and countering the currently bleak prognosis. The crux of personalized medicine lies in the early diagnosis and staging of lung cancer, alongside predicting its pathological subtypes. Despite needle biopsy retaining its status as the diagnostic gold standard for lung cancer, its invasiveness, low reproducibility, potential for false negative outcomes, and risk of complications have spurred the need for improved methods (5). In this regard, medical imaging technology, particularly low-dose computed tomography (CT), was recommended for screening high-risk lung cancer populations by the 2011 National Lung Screening Trial and the 2020 Dutch-Belgian Lung Cancer Screening Trial. This approach aims to elevate early diagnosis rates and mitigate mortality (6). However, the use of low-dose CT screening carries the risk of false positive results, erroneously identifying non-cancerous pulmonary nodules as lung cancer, thus complicating clinical diagnoses. In recent years, imaging radiomics has gained prominence, especially the application of PET-related techniques like positron emission tomography (PET)/CT and PET/magnetic resonance imaging (MRI) radiomics. These techniques not only integrate insights into functional metabolism and anatomical structure insights but also enable facilitate comprehensive, single-session examinations. This proves invaluable for various aspects of lung cancer, including diagnosis, treatment, prediction of pathological subtypes, evaluation of systemic metastasis evaluation, disease staging, and assessment of treatment efficacy assessment. In light of this, the present manuscript aims to delve into the utility of PET-related traditional imaging methods in lung cancer, the research surrounding the application of PET/CT and PET/MRI radiomics in lung cancer diagnosis and treatment, AI-based analysis of PET-related radiomics, as well as the challenges and prospects of PET-related radiomics.

## 2 Application and development of PET-related traditional imaging techniques in lung cancer

In the realm of emerging imaging technologies, PET, PET/CT, and PET/MRI are key tools for assessing tumor metabolism, evaluating treatment outcomes. A prospective study by Kirchner et al. (7) involving 84 NSCLC patients found no significant difference in the precision of tumor (T) and node (N) staging between PET/CT and PET/MRI in a prospective study involving 84 NSCLC patients. Martin et al. (8) similarly established the comparability of PET/MRI and PET/CT in staging lung cancer, substantiated by imaging results from 1003 non-small cell lung cancer (NSCLC) patients. Additional studies by Dahlsgaard-Wallenius (9), Mayerhoefer (10), and Kim (11) emphasized the enhanced value of PET/MRI over PET/CT in detecting metastases, particularly in the brain, adrenal glands, liver, and bone marrow. Notably, PET/MRI showed significantly improved specificity in identifying metastatic sites. The incorporation of bone marrow-specific sequences further enhanced the comprehensiveness of lung cancer staging or restaging. Recent studies have explored the metabolic aspects of lung cancer. Chandarana et al. (12) examined pulmonary nodules and closely correlated PET/MRI with PET/CT’s maximum standardized uptake value (SUVmax) measurements. PET/MRI’s mean SUVmax was 16.4% ± 13.6% higher than that of PET/CT. Kohan et al. (13) delved into fluorodeoxyglucose (FDG) avid lymph node SUVmax in lung cancer patients, revealing a 27.27% higher value in PET/MRI (5.85) compared to PET/CT imaging (4.60). This approach also demonstrated a strong correlation (r = 0.93) and improved efficacy in detecting metastases in regions like the brain, adrenal glands, and bone marrow. PET/MRI excels at identifying metastases with minimal metabolic activity, which may go unnoticed using PET/CT. While PET/MRI exhibits 96% sensitivity for FDG-avid nodules and 89% sensitivity for nodules measuring ≥ 5 mm, its effectiveness diminishes for smaller nodules (< 4 mm), with a sensitivity of 38%. However, Raad et al. (14) observed that many nodules measuring less than 5 mm missed in PET/MRI, either regressed or remained stable, suggesting their benign nature. Yi et al. (15) highlighted PET/MRI’s advantages in detecting malignant soft tissue tumors (e.g., brain, bone, muscle, head, neck, breast, and liver primaries) and lymph node metastasis. The enhanced sensitivity in NSCLC patients improved preoperative lymph node staging. PET/MRI also enhanced tumor classification and staging, thanks to thoracic MRI’s superior tumor delineation and mediastinal extent detection compared to CT in PET-CT. Advanced MRI techniques added value by providing molecular imaging insights, including MR spectroscopy, diffusion-weighted MR, and perfusion imaging, all without subjecting patients to additional radiation exposure (16). While conventional imaging remains the standard for lung cancer screening, it falls short in quantifying tumor heterogeneity, exhibiting limited reproducibility and stability. To overcome these limitations, radiomics has gained prominence. By using automated data characterization algorithms, radiomics transforms region-of-interest image data into comprehensive spatial data. This process translates measurements related to the behavior of unobservable cells and molecules into high-dimensional information, enhancing diagnostic, monitoring, and prognostic capabilities for clinical practice.

## 3 Progress in the application of PET-related radiomics in lung cancer

PET/CT and PET/MRI, molecular hybrid imaging methods, offer simultaneous anatomical, metabolic, and functional insights, coupled with whole-body scanning capabilities. Radiomics based on PET/CT and PET/MRI combine morphoanatomical, functional, and/or metabolic details. This approach facilitates quantitative or semi-quantitative analysis of internal tumor heterogeneity and metabolic characteristics. It introduces a novel perspective and technique for the early detection and characterization of tumors, including their molecular traits. In recent years, there has been a surge in studies exploring the utility of PET-related radiomics in various aspects of lung cancer, including diagnosis, prediction of pathological subtype prediction, assessment of metastasis, receptor and molecular forecasts, as well as prognosis and treatment efficacy evaluations (17–57).

### 3.1 PET-related radiomics in the diagnosis of lung cancer

Effective disease management and prognosis in lung cancer rely on early diagnosis. The role of PET-related radiomics in lung cancer diagnosis has significantly expanded. Liu et al. (17) leveraging PET/CT radiomics features, effectively distinguished peripheral lung cancer from inflammatory pseudotumor. In a retrospective study involving 545 lung cancer patients, Kirienko et al. (18) demonstrated the potential of PET textural features to differentiate primary lung cancer from metastatic tumors, and to identify primary lung cancer’s pathological subtypes. Kang et al. (19) developed a hybrid nomogram that incorporated PET/CT radiomics features and manual diagnosis, resulting in reduced false positive rates (FPR), enhanced diagnostic accuracy, and improved net clinical benefits. Notably, PET/CT radiomics have shown superiority over conventional PET/CT in distinguishing primary tumors from metastatic tumors, assessing tumor recurrence after radiotherapy, and detecting radiation-induced inflammatory reactions (18, 20, 21).

### 3.2 PET-related radiomics predicts different pathological subtypes of lung cancer

The inherent heterogeneity of lung cancer necessitates distinct treatment approaches, highlighting the importance of early identification of pathological subtypes for tailored precision therapy. PET/CT radiomics have demonstrated the capability to differentiate between lung adenocarcinoma and lung squamous cell carcinoma. Furthermore, the disparities in PET radiomics traits between these subtypes vary across different stages (20–25). Effective models based on PET/MRI images have also been established for predicting lung adenocarcinoma versus squamous cell carcinoma (2, 26). Meng et al. (27) developed a model for predicting noninvasive small cell lung cancer (SCLC) versus NSCLC, alongside distinguishing epidermal growth factor receptor (EGFR) mutation type and wild-type NSCLC, based on PET/MRI images, amide proton transfer weighted imaging (APTWI), and diffusion-weighted imaging (DWI) in 99 lung cancer patients. Zhou (28) demonstrated that ^18^F-FDG PET/CT radiomics features, in conjunction with machine learning techniques, can differentiate primary from metastatic lung lesions and identify lung cancer’s histological subtypes. Dunn et al. (29) emphasized the potential of artificial intelligence-based computer-aided diagnostic tools, integrating radiomics analysis image segmentation with supervised classification, to autonomously diagnose lung cancer subtypes. Shen et al. (30) effectively classified lung adenocarcinoma (ADC) and squamous cell carcinoma (SCC) by employing subregion-based radiomics features extracted from ^18^F-fluorodeoxyglucose (^18^F-FDG) PET/CT images of 150 ADC and 100 SCC patients. The all studies (**Table 1**) indicated that PET related radiomics had significant significance in non-invasive and conveniently prediction of pathological subtypes of lung cancer. So then, PET related radiomics can assist clinical decision-making earlier.

**Table 1:** The references collection of PET-related radiomics predicting different pathological subtypes of lung cancer.

Author	Cases	Type of model	Imaging methods	Verification	Classifiers	Model evaluation	Result Optimal model (AUC values)
Xin Tang (2)	80	combined	PET/MR CT	10-fold cross-validation	GP	9 models	PET/MRI + CT + Clinical model (0.965)
Xin Tang (26)	61	single	PET/MR	5-fold cross-validation	mRMR, LASSO	1 model	PET/MRI radiomics model (0.886)
Nan Meng (27)	99	combined	PET/MR	NA	Logistic regression	6 models	Combination of MTV, ADC, and MTRasym model (0.953)
Yi Zhou (28)	769	combined	PET/CT	10-fold cross-validation	SVM, LDA, DT, RF, KNN, GBDT, AdaBoost, LR, GaussianNB	45 models	Combination of GBDT feature selection method with GBDT classification model (0.897)
Bryce Dunn (29)	355	combined	PET/CT	5-fold cross-validation	decision tree, discriminant, naïve Bayes, support vector machine, k-nearest neighbors, ensemble, narrow neural network	7 models	Support vector machine model (0.97)
Hui Shen (30)	250	combined	PET/CT	5-fold cross-validation	SVM linear, SVM RBF, RF, LR, GP, Lincar discriminant, Adaboost classifier	4 models	PET/CT radiomics with SVM-RBF classification model (0.9155)

AUC, Area under the curve; GP, Gaussian process; RMR, Maximum relevance and minimum redundancy; LASSO, Least absolute shrinkage and selection operator; NA, Not available; MTV, Metabolic tumor volume; ADC, Apparent diffusion coefficient; MTRasym, Magnetization transfer ratio asymmetry; GBDT, Gradient boosting decision tree; SVM, Support vector machine; RBF, Radial basis function; LDA, Linear discriminant analysis; DT, Decision tree; RF, Random forest; KNN, K-nearest neighborhood; AdaBoost, Adaptive boosting; LR, Logistic regression; GaussianNB, Gaussian Naive Bayes.

### 3.3 PET-related radiomics assessment of metastasis and lung cancer stage

Lung cancer, one of the most lethal malignancies (1, 31), constitutes over 85% of NSCLC cases. For NSCLC patients, surgical resection is the primary treatment, significantly improving prognosis. However, those with lymph node or distant metastases require systematic lymph node dissection and additional metastatic treatment alongside lesion resection (32, 33). These comprehensive approaches markedly enhance patient outcomes. Therefore, precise and early assessment of metastasis holds profound implications for lung cancer prognosis. Alongside CT and MRI radiomics, PET-related radiomics play a crucial role in evaluating metastasis. Giesel et al. (34) utilized volumetric CT histograms, in conjunction with PET/CT radiomics models, to analyze CT values of lymph nodes in 148 lung cancer patients, totaling 1022 nodes. The study integrated their SUVmax values and identified lymph node density as a crucial parameter for distinguishing between benign and malignant nodes. Huang et al. (35) predicted mediastinal lymph node metastasis in 155 NSCLC patients using ^18^F-FDG PET/CT imaging radiomics. Similarly, Ouyang et al. (36) demonstrated that models incorporating 18F-FDG PET/CT and CT images effectively predicted mediastinal-hilar lymph node metastasis (LNM) true and false positives in NSCLC patients. This insight aids clinicians in formulating personalized treatment strategies. Comparing PET/MRI radiomics staging with CT radiomics staging, Kajiyama et al. (37) observed better consistency with pathological staging in PET/MRI radiomics staging (59/82 cases). PET/MRI, CT, and pathological staging (stage I or less vs. stage II or more) revealed distinctions as prognostic factors for recurrence or metastasis. Preoperative PET/MRI radiomics staging demonstrated superior survival prediction, surpassing CT prediction models in diagnosing hilar and mediastinal lymph node metastasis. Zhang et al. (38) confirmed the effectiveness of ^18^F-FDG PET/MRI radiomics in forecasting pleural invasion in lung adenocarcinomas smaller than 3 cm. These studies showed that PET-related radiomics was highly effective in predicting lung cancer stage and metastasis of whole body. Although histopathology is the gold standard for diagnosis of lung cancer, it is invasive, and the sample is local which may lead to false negative results. However, PET-related radiomics can dynamically and comprehensively reflect the tumor features and metastasis of the patients with lung cancer, and it can reflect the tumor metabolism, which is noninvasive and reproducible.

### 3.4 PET-related radiomics predicts receptors and molecular tumor microenvironment

As immunotherapy gains momentum in cancer treatment, understanding an individual’s tumor immune microenvironment (TIME) phenotype, especially the tumor immune types (TMITs) associated with immunotherapy effectiveness, becomes crucial. Profiling the TIME phenotype aids in patient selection for immunotherapy. Agüloğlu (39) and Chang (40) predicted anaplastic lymphoma kinase (ALK) rearrangement and EGFR mutation status in NSCLC via radiomics models based on ^18^F-FDG PET/CT images, assisting in targeted therapy decisions. Numerous studies have demonstrated the capability of ^18^F-FDG PET/CT radiomics models to predict programmed death ligand-1 (PD-L1) expression in NSCLC (41–44). Aide et al. (45) developed a model based on clinical features and pretreatment ^18^F-FDG PET images to detect alterations in key molecular targets in lung adenocarcinoma, opening new possibilities for patient selection for molecular targeted therapy. Tong et al. (46), analyzing data from 1145 NSCLC patients, demonstrated the superiority of PET/CT radiomics in predicting CD8 expression compared to the CT model (area under the curve (AUC): 0.907 vs. 0.861, *P* = 0.0314). Moreover, the combined PET/CT radiomics-clinical model (AUC = 0.932) outperformed the PET/CT radiomics model (AUC = 0.907, *P* = 0.0326) and the clinical model (AUC = 0.868, *P* = 0.0036) in predicting CD8 expression. Zhou et al. (47) similarly demonstrated the efficacy of ^18^F-FDG-PET/CT-based radiological features in predicting TMIT-I in NSCLC, offering a promising approach for immunotherapy selection. Meng et al. (48) conducted a prospective collection of ^18^F-FDG PET/MRI image data from 76 NSCLC patients to develop a predictive model. Their findings highlighted the value of ^18^F-FDG PET/MRI for the non-invasive assessment of PD-L1 status in NSCLC. The combination of magnetization transfer rate asymmetry (MTRasym 3.5 ppm), diffusion coefficient (D), and SUVmax at 3.5 ppm exhibited robust predictive capacity for PD-L1-positive and PD-L1-negative NSCLC (AUC: 0.946 (0.869 – 0.985); sensitivity: 0.853; specificity: 0.916; *P* < 0.001). Receiver operating characteristic curve (ROC) and calibration curves confirmed the accuracy and consistency of their findings.

### 3.5 Prognostic efficacy assessed by PET-related radiomics

Lung cancer treatment is intricate, involving challenging prognostic predictions. Due to the patient-tumor heterogeneity, achieving a balance between clinical efficacy, side effects, and costs necessitates personalized and adaptable treatment approaches. Current clinical practice often relies on genomic and proteomic techniques to analyze tumor biological traits, guiding tailored treatments. However, these techniques often involve invasive procedures and offer limited real-time and comprehensive insights due to the temporal-spatial heterogeneity of tumors. In contrast, radiomics, particularly PET-related radiomics, offers non-invasive and repeatable advantages. It combines metabolic function insights, gaining traction for real-time prognosis monitoring in cancer treatment. Hoekstra et al. (49), utilizing ^18^F-FDG PET/CT imaging before and after chemotherapy in 47 non-small cell lung cancer patients, identified a 50% or more decrease in standardized uptake value (SUV) values as an indicator of a favorable prognosis. Christie et al. (50) developed a predictive model based on postoperative CT and PET/CT images of 135 NSCLC patients, stratifying patients into low and high recurrence/progression risk groups. Preoperative ^18^F-FDG PET/CT radiomics, as discovered by Onozato et al. (51) could predict highly invasive lung cancer. Kirienko et al. (52) revealed that ^18^F-FDG PET/CT radiomics features predicted postoperative NSCLC recurrence, positioning these features as useful biomarkers for prognosis and risk stratification enhancement. Yang et al. (53) and Mattonen et al. (54) established models combining ^18^F-FDG PET/CT images with clinicopathological data, successfully assessing overall the survival rate and enhancing NSCLC recurrence prediction. Dissaux et al. (55) identified two features from an ^18^F-FDG PET/CT image model that independently associated with local control in NSCLC patients receiving SBRT, providing insights into local recurrence and aiding clinical decisions. Ahn et al. (56) constructed a PET-based imaging model with radiomics features, aiding risk stratification and validating NSCLC recurrence risk classification through machine learning methods. Kirienko et al. (57) further demonstrated that CT, PET, or PET/CT image-based model characteristics could predict disease-free survival (DFS) in surgically treated NSCLC patients.

## 4 AI-based analysis of PET-related radiomics

**Figure 1:**
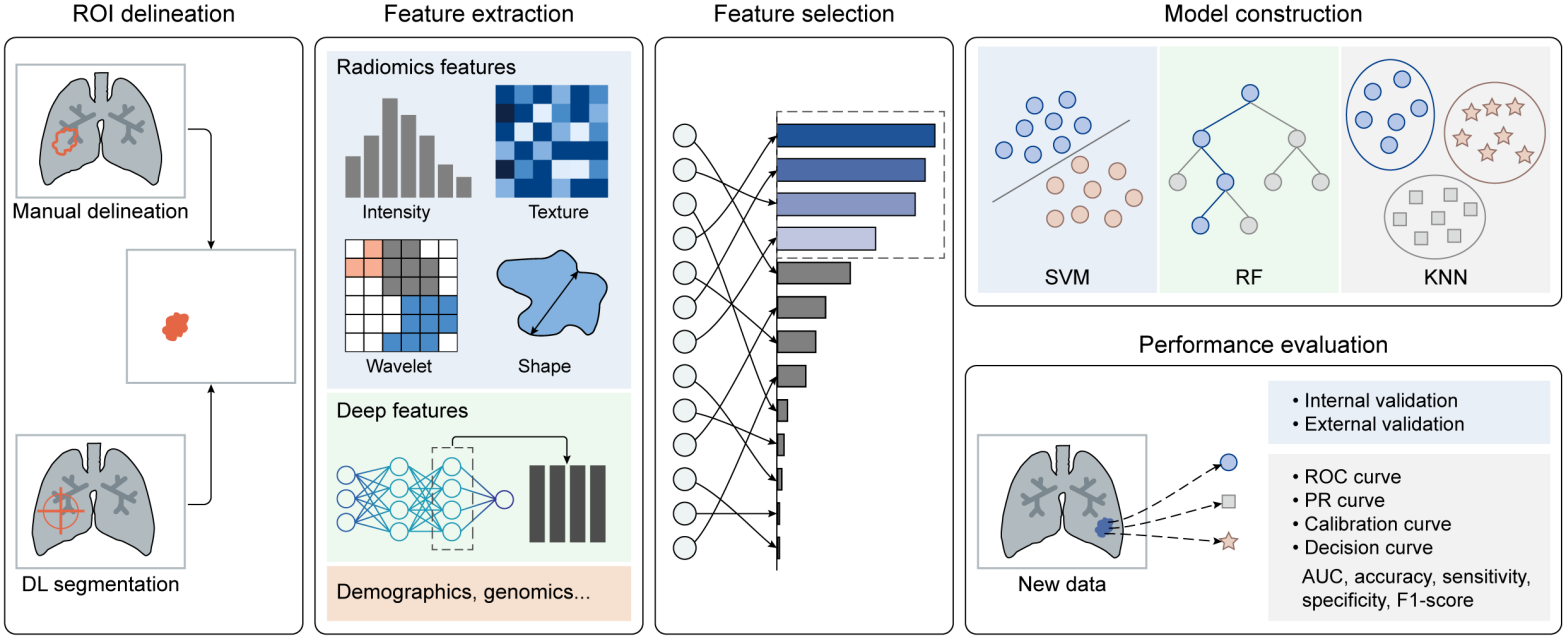
Artificial intelligence (AI)-based radiomics analysis workflow.

Artificial intelligence (AI), represented by machine learning (ML) and deep learning (DL) algorithms, can discern patterns and relationships in training data and employ these insights to make predictions about new data. There are two primary types of AI: supervised learning and unsupervised learning. Supervised learning uses labeled data to train models that establish mappings between features and categories, whereas unsupervised learning uncovers natural groupings or categories within unlabeled data. AI-based radiomics involves extracting quantitative features from extensive medical images and constructing predictive models that correlate image features with clinical endpoints, all of which fall under supervised learning. The radiomics workflow can be divided into five parts (as depicted in **Figure 1**): [1] image labeling, [2] feature extraction, [3] feature selection, [4] model construction, and [5] performance evaluation. Traditional ML algorithms are involved in [2] through [5], while the DL strategy primarily plays a role in [1] and [2], offering potential to enhance the automation and efficiency of radiomics analysis.

### 4.1 ROI delineation

The initial and most critical step is to accurately identify the region of interest (ROI), typically displaying morphological or metabolic abnormalities. Manual delineation is often considered the gold standard, but it is burdened by being laborious, time-consuming, and prone to errors, making it challenging to achieve high-quality and efficient labeling. In light of this, semi-automatic and fully-automatic methods based on DL segmentation algorithms have emerged. These methods not only maintain high segmentation accuracy but also significantly enhance efficiency. It’s worth noting that PET imaging is affected by a partial volume effect due to its relatively low spatial resolution. This effect results in the merging of multiple small nodules into a single large nodule or the segmentation of one large nodule into multiple smaller nodules, compromising the accuracy of DL segmentation algorithms. To address this issue, a registration algorithm can be employed to align the functional PET image with the structural image (i.e., MR or CT). Utilizing anatomical information from MR or CT images allows for effective localization and segmentation of lung nodules in PET images, improving accuracy in lung nodule segmentation.

Furthermore, various processes like dilation and erosion can be applied to manual, semi-automatic, and fully-automatic ROIs to focus on the most significant region. Notably, splitting the dataset is necessary for the subsequent evaluation of model performance, where the model is trained on the training dataset and evaluated on the testing dataset. Since radiomics features are sensitive to variations in gray level, pixel size, and slice thickness of images, image preprocessing (e.g., normalization and resampling) should be performed before calculating radiomics features (58–61).

### 4.2 Feature extraction

After image preprocessing, high-throughput radiomics features can be automatically extracted from each ROI (in the PET, CT, and MR) in accordance with the guidelines of the imaging biomarker standardization initiative (IBSI) (62). The commonly used features fall into three categories: [1] first-order statistics describing voxel intensity; [2] shape-based features reflecting region shape and size; and [3] textural features, including gray level co-occurrence matrix (GLCM) features, gray level run length matrix (GLRLM) features, gray level size zone matrix (GLSZM) features, neighboring gray-tone difference matrix (NGTDM) features, and gray levels dependent matrix (GLDM) features. These textural features quantify regional heterogeneity differences. Additionally, multiple filters can be applied to the original image to enhance feature richness. Moreover, other factors such as handcrafted features, end-to-end deep features from DL models, demographics, biochemical information, and genomics may also be incorporated to construct a predictive model.

### 4.3 Feature selection

Given the high-throughput nature of radiomics features, it is essential to perform feature selection or dimensionality reduction to identify robust imaging biomarkers. Feature selection involves assessing the importance or relevance of features to the prediction task and selecting a subset from the original set. This can be done using methods such as the least absolute shrinkage and selection operator (LASSO), variance thresholding, and SelectKBest. Dimensionality reduction aims to transform the high-dimensional feature space into a lower-dimensional one while preserving as much information as possible. Techniques like linear discriminant analysis (LDA) and principal component analysis (PCA) are used for this purpose. Features selected through feature selection provide interpretability, while features obtained through dimensionality reduction capture important patterns.

### 4.4 Model construction

Based on the selected features, various ML-based models can be developed for different tasks. Representative ML algorithms, such as logistic regression (LR), support vector machine (SVM), random forest (RF), extreme gradient boosting (XGBoost), decision tree (DT), and k-nearest neighbor (KNN), are widely employed for medical image classification and regression tasks. These classifiers are trained on a training dataset to determine the most suitable parameters for mapping features to categories or specific values. It’s noteworthy that compared to traditional multi-parameter prediction methods, such as PCA, ML algorithms offer several advantages. Firstly, they have the ability to capture the complex nonlinear relationships between input and target variables. Additionally, they can be optimized based on labeled data, resulting in higher prediction accuracy. Lastly, they are applicable to various problem types and data domains, demonstrating superior generalization capabilities.

### 4.5 Performance evaluation

Model performance should be assessed using internal or external validation datasets. Multiple quantitative metrics can be calculated to evaluate the model’s performance.

For classification tasks, it is recommended to plot receiver operating characteristic (ROC) curves to visualize the trade-off between the true positive rate and false positive rate at various classification thresholds. The area under the curve (AUC) is often calculated to quantify the overall performance of the model. Additionally, metrics like accuracy, sensitivity, specificity, and F1-score can also be computed. Calibration curves assess how well predicted probabilities from a model align with observed outcomes. Decision curves help researchers determine whether radiomics-based predictive models would improve clinical benefits compared to treating all or none of the patients.

For regression tasks, common metrics include mean squared error (MSE), mean absolute error (MAE), R-squared, and the Pearson correlation coefficient. A combination of multiple evaluation metrics and rigorous statistical analysis can provide a comprehensive assessment of model performance for regression tasks. In the realm of AI-based radiomics analysis, an increasing number of software tools have been developed to streamline and automate the process of extracting, analyzing, and interpreting radiomics features from medical images. Representative software includes Pyradiomics (http://pyradiomics.readthedocs.io/en), Radiomics Toolbox (https://www.radiotoolbox.com/), IBEX (Imaging Biomarker Explorer, http://bit.ly/IBEXSrc_MDAnderson), and uAI Research Portal (https://www.uii-ai.com/en/uai/scientific-research), which aim to improve the automation, standardization, reproducibility, and efficiency of radiomics analysis.

## 5 Challenges and prospects of PET-related radiomics

In recent years, AI-based radiomics analysis has made remarkable progress in lung cancer assessment. However, several challenges and prospects of PET-related radiomics need to be addressed before its widespread use in clinical practice.

### 5.1 Data scarcity and heterogeneity

The development of automated clinical solutions, such as radiomics analysis, faces a significant challenge in obtaining high-quality and large-scale data. Pathologically confirmed ground truth typically relies on invasive surgical resection or needle biopsy, while manual annotation is time-consuming and costly, requiring experienced radiologists with extensive domain knowledge. To address this issue, the creation of open-source image repositories through cross-institutional data sharing has proven effective in increasing data volume. However, variations in institutions, scanners, acquisition protocols, and image post-processing algorithms can introduce significant heterogeneity into the images. DL-based approaches show promise in improving data quality and quantity through data augmentation and reducing the dependency on manual annotation through unsupervised learning. Efforts towards standardization are needed to minimize data heterogeneity and improve model generalizability.

### 5.2 Model performance

Class imbalance presents a significant challenge to model performance. Notably, the majority of lung nodules are benign (58). AI systems trained on imbalanced datasets struggle to adequately learn valuable features from minority classes and tend to treat all items as a single majority class. To address these problems, methods such as data augmentation and data generation have been developed to offer potential benefits. Additionally, models trained on specific datasets often demonstrate excellent performance for specific tasks but may not maintain the same level of performance in more general situations. This lack of generalizability makes it challenging to assess the overall applicability of trained models. Furthermore, it is essential to note that most AI algorithms are developed retrospectively and lack prospective validation. Prospective validation trials should be conducted with diverse patient populations to provide a realistic assessment of the clinical utility of AI.

### 5.3 Model interpretability

There is a lack of transparency regarding the inner workings of AI methods, leaving clinicians with a ‘black box’ when reviewing AI-generated results. To enhance clinicians’ confidence in AI-informed decisions, it is crucial to emphasize the interpretability and transparency of AI models. Data visualization tools can aid in understanding how algorithms arrive at their decisions to some degree. For instance, class activation maps can highlight the areas that algorithms prioritize during the decision-making process (63).

### 5.4 Ethical issue

From an ethical standpoint, several issues need to be addressed before considering AI systems as viable decision-making tools. These issues encompass determining accountability for incorrect AI decisions, evaluating public perception of AI decision tools, and addressing concerns regarding data security and privacy. Furthermore, there should be restrictions on over-reliance on automation and the disregard for common sense.

## Author contributions

XT: Writing – original draft, Writing – review & editing. FW: Writing – review & editing. XC: Writing – original draft. SY: Writing – original draft. ZD: Writing – original draft, Writing – review & editing.
